# Mature but not developing Schwann cells promote axon regeneration after peripheral nerve injury

**DOI:** 10.1038/s41536-022-00205-y

**Published:** 2022-01-28

**Authors:** Takeshi Endo, Ken Kadoya, Tomoaki Suzuki, Yuki Suzuki, Mohamad Alaa Terkawi, Daisuke Kawamura, Norimasa Iwasaki

**Affiliations:** Department of Orthopaedic Surgery, Faculty of Medicine and Graduate School of Medicine, Hokkaido University, Kita-15 Nishi-7, Sapporo, Hokkaido 060-8638 Japan

**Keywords:** Regeneration and repair in the nervous system, Translational research, Somatic system

## Abstract

Since Schwann cells (SCs) support axonal growth at development as well as after peripheral nerve injury (PNI), developing SCs might be able to promote axon regeneration after PNI. The purpose of the current study was to elucidate the capability of developing SCs to induce axon regeneration after PNI. SC precursors (SCPs), immature SCs (ISCs), repair SCs (RSCs) from injured nerves, and non-RSCs from intact nerves were tested by grafting into acellular region of rat sciatic nerve with crush injury. Both of developing SCs completely failed to support axon regeneration, whereas both of mature SCs, especially RSCs, induced axon regeneration. Further, RSCs but not SCPs promoted neurite outgrowth of adult dorsal root ganglion neurons. Transcriptome analysis revealed that the gene expression profiles were distinctly different between RSCs and SCPs. These findings indicate that developing SCs are markedly different from mature SCs in terms of functional and molecular aspects and that RSC is a viable candidate for regenerative cell therapy for PNI.

## Introduction

In contrast to the poor regenerative capacity of the central nervous system (CNS), the peripheral nervous system can regenerate after injury. However, the clinical outcome is not always satisfactory especially in case of proximal injury or large defect^1,2^. In addition, autologous nerve graft (ANG) has been a gold standard since the early 20th century for the reconstruction of nerve injury with large defects,^1,3,4^, still accompanying several issues such as donor site morbidity, limited supply, and motor-sensory and size mismatch^4,5^. Due to the recent advancement of biomaterials, new treatment options such as a synthetic scaffold or decellularized allograft became clinically available to overcome the issues derived from ANG^6,7^. However, their axon-promoting effects are still not comparable to ANG. Accordingly, a therapy superior to ANG has been desired for decades.

Recently, cell therapy has been attracting much attention as a potent therapy in multiple organs^8–13^. Considering a cell type for the graft for peripheral nerve injury (PNI), peripheral glia, Schwann cell (SC), is a potent candidate because the graft of SCs enhanced axon regeneration after PNI^14–20^ and regenerating axons always accompany SCs^21^. Further, with the rapid growth of stem cell research, novel SC-like cells have been developed as a new graft material. They have differentiated from various kinds of stem cells^22–26^ or fibroblasts with direct reprogramming technique^27^.

Interestingly, the SCs used for the study of axon regeneration after PNI has been always mature cells, and developing SCs were rarely tested, although they involve axonal development and finally differentiate into mature SCs^28^. This is a sharp contrast to the study of CNS injury where developing but not mature neural cells are the main cell types used for therapeutic purposes including axon regeneration^10,29,30^. Accordingly, it is reasonable to assume that the SCs at developmental stages can also promote axon regeneration or might be superior to mature SCs as a graft cell source for PNI.

Importantly, it was previously thought that SCs dedifferentiated into the phenotype of developing SCs after injury to support the reparative process, based on the observation that so many gene expressions of denervated nerves were shared with developing nerves, including loss of myelin and upregulation of developing SC markers^31–33^. Recently, accumulated evidence replace this idea with a new one that injury response reprograms mature SCs and converts them to repair SCs (RSCs) specializing in the repair process and that they are substantially different from developing SCs^28,31^. However, this recent evidence does not necessarily deny the reparative potential of developing SCs. Further, it remains unclear about the actual difference of the molecular profiles between RSCs and developing SCs.

Therefore, the purpose of the current study is to elucidate the therapeutic potential of developing SCs by investigating axon-promoting effects after PNI and by clarifying their molecular profiles, compared to RSCs. The current study demonstrates that, unlike CNS, developing SCs have no capacity to support the regeneration of adult axons after PNI, that developing SCs are distinctly different from RSCs in molecular and functional aspects, and that RSC is a potent candidate as a graft cell type for axon regeneration therapy after PNI.

## Results

### Mature SCs but not developing SCs supported axon regeneration

**Fig. 1:**
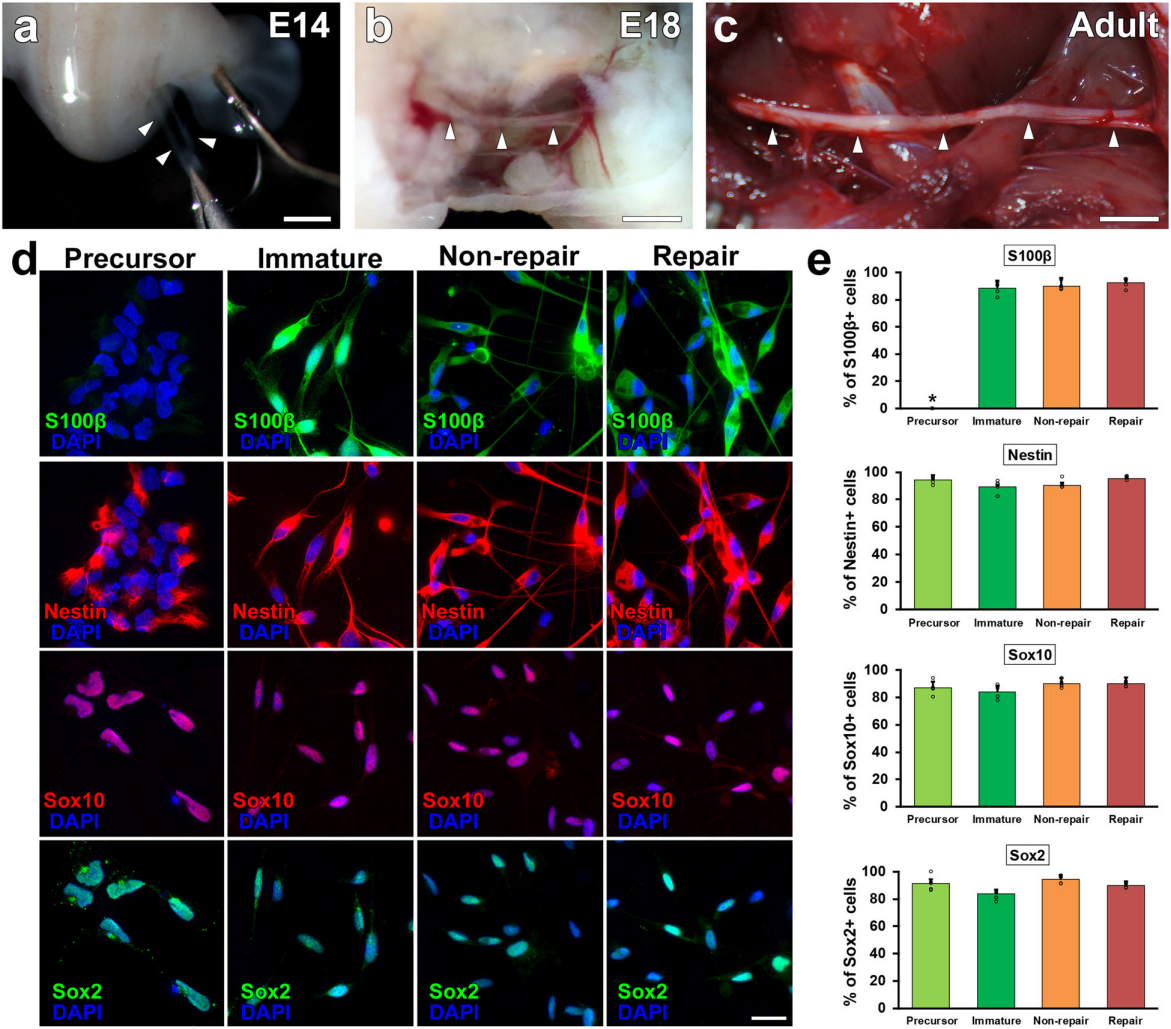
Characteristics of prepared SCs. **a**–**c** Outlook of sciatic nerves at different developmental stages. SCPs, ISCs, and non-RSCs were harvested from intact sciatic nerves at E14 (**a**), E18 (**b**), and postnatal 10–12 weeks (**c**). Arrowheads indicate sciatic nerves. Scale bars: 1 mm (**a**, **b**), 5 mm (**c**). **d** Immunolabeling of cultured SCs against S100β, Nestin, Sox10, and Sox2. Regardless of SC types, most cells express immature marker, Nestin and Sox2, and SC marker, Sox10. In contrast, another SC marker, S100β is absent only in SCPs. Scale bars: 20 µm. **e** Quantifications of % of immunolabeled SCs against S100β, Nestin, Sox10, and Sox2. Five samples per group. At least 50 cells were counted in each sample. *P < 0.05 vs. SCP, one-way ANOVA with the Tukey–Kramer test. Error bars represent the SEM.

SCs develop from neural crest cells, differentiate to SC precursors (SCPs) around E14 (Fig. 1a) and then to immature SCs (ISCs) around E18 (Fig. 1b)^34,35^. Accordingly, we tested the axon-promoting effect of SCPs and ISCs by comparing two types of mature SCs, RSCs, and nonRSCs prepared from injured and intact nerves, respectively (Fig. 1c). Characterization of prepared SCs demonstrated that SC marker, Sox10, was expressed in all types of SCs but another SC marker, S100β, is not expressed in SCPs (Fig. 1d)^36–38^. Based on Sox10 immunolabeling, the purity of prepared SCs were 87%, 95%, 95%, and 92% for SCPs, ISCs, nonRSCs, and RSCs, respectively (Fig. 1d, e). When examining immature markers, not only developing but also mature SCs express nestin and Sox2 (Fig. 1d, e, Supplementary Fig. 1), suggesting that dissociated SCs start expressing these markers^39–41^.

**Fig. 2:**
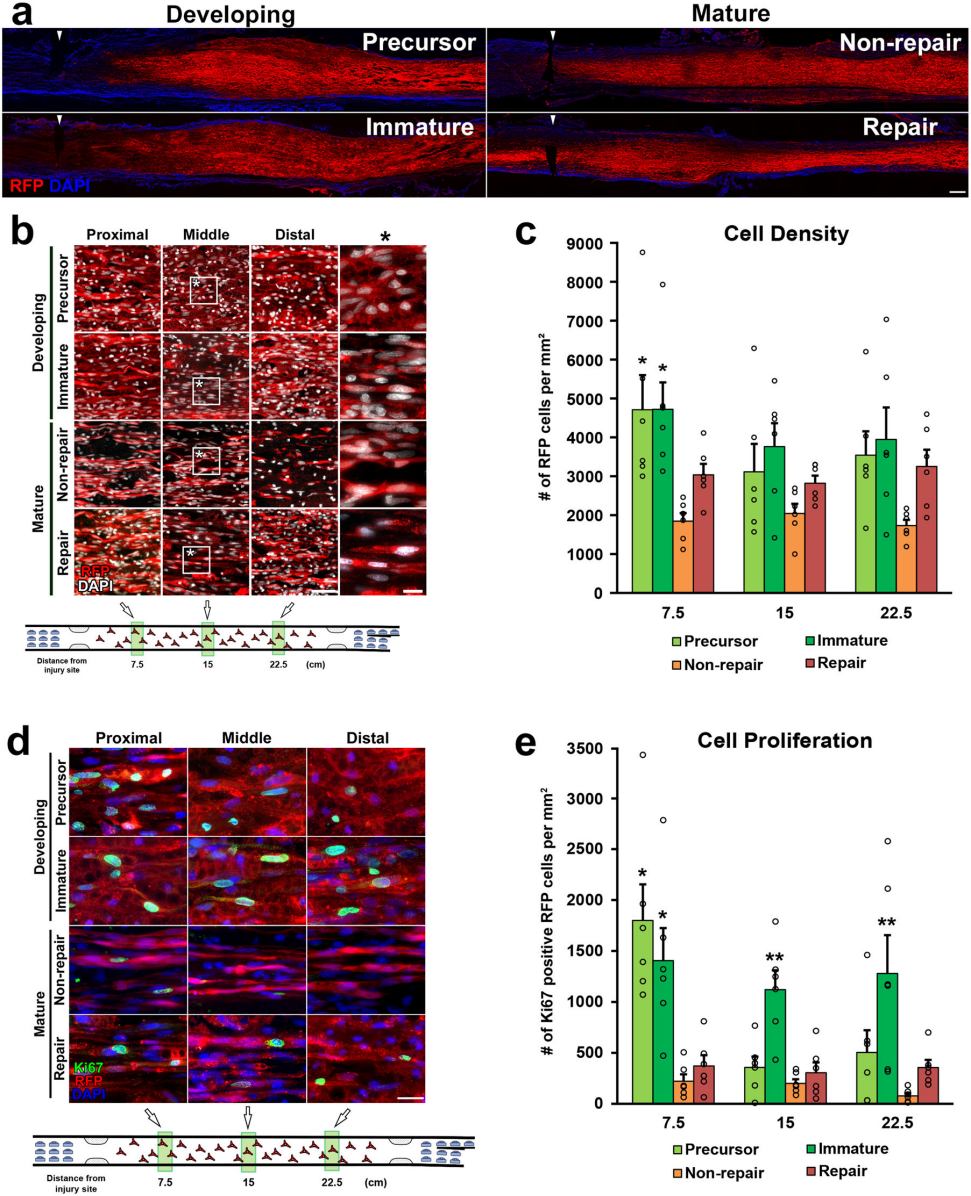
Cell density and proliferative property of grafted SCs. **a** Low magnification images of longitudinal sections around the proximal injury sites 2 weeks after SCs grafts. RFP expressing SCs of all types fulfill injured nerves. Arrowheads indicate the proximal injury sites marked by small cuts. Left is proximal. Scale bar, 500 µm. **b** RFP expressing grafted SCs at proximal, middle, and distal one-third of graft areas. Scale bar, 100 µm. *: High magnification image of the boxed area. Scale bar, 10 µm. **c** Quantification of the density of grafted cells at 7.5, 15, and 22.5 mm points from the proximal injury site. At the 7.5 mm point, SCPs and ISCs graft subjects demonstrate statistically higher densities of RFP expressing cells than RSCs and non-RSCs subjects. Six rats per group. *P < 0.05 vs. n RSC and non-RSC, One-way ANOVA with the Tukey–Kramer test. Error bars represent the SEM. **d** High magnification images of Ki67 immunolabeling at the proximal, middle, and distal one-third of graft areas. Scale bar, 20 µm. **e** Quantification of the density of Ki67 immunolabeled RFP expressing graft cells at 7.5, 15, and 22.5 mm points from the proximal injury site. Developing SCs have more proliferating than mature SCs after grafting. *P < 0.05 vs. non-RSC and RSC, **P < 0.05 vs. other 3 groups. One-way ANOVA with the Tukey–Kramer test. Error bars represent the SEM.

**Fig. 3:**
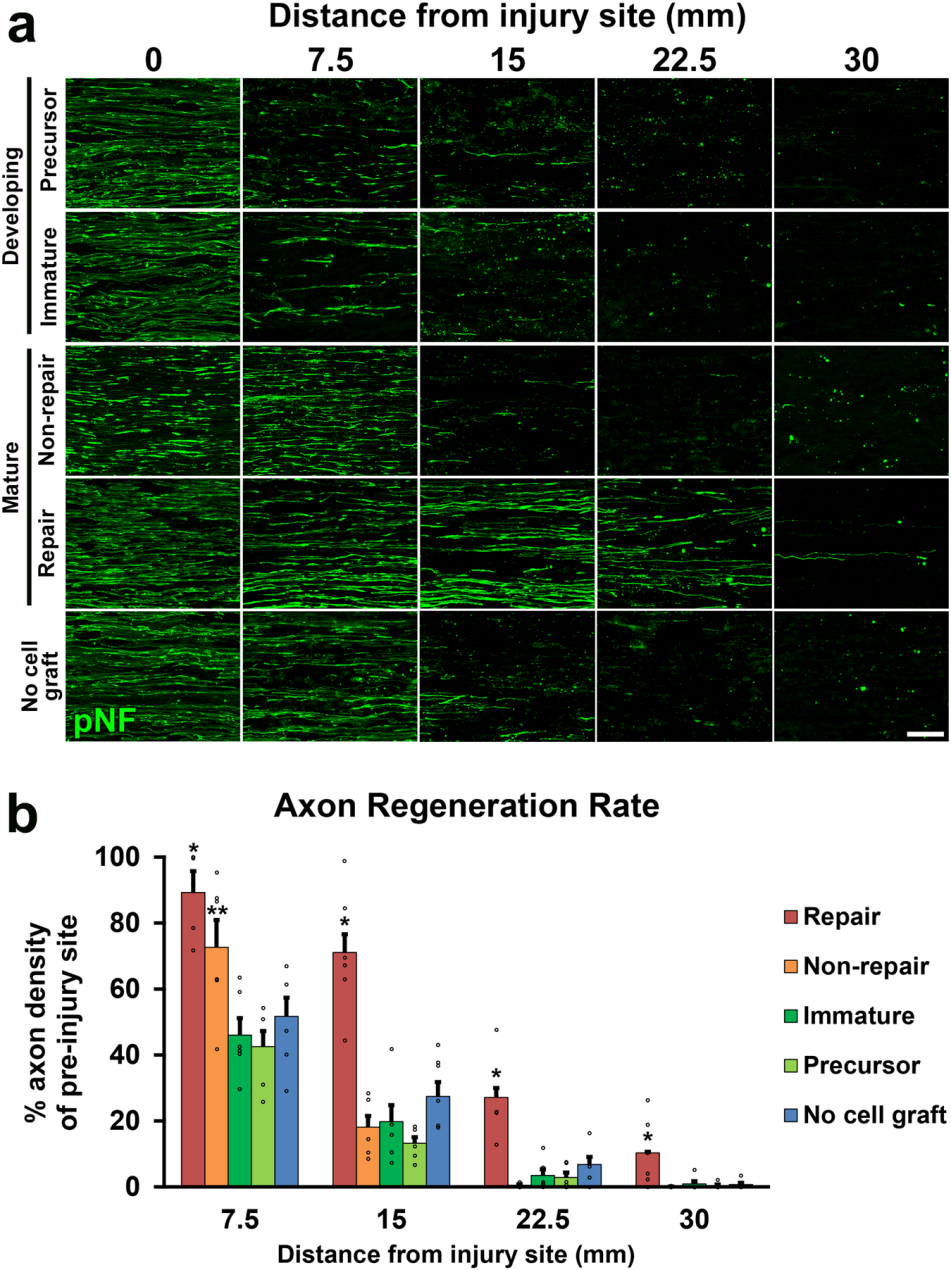
Axon regeneration induced by grafted SCs. **a** Representative images of regenerating axons labeled by pNF in five groups, which are graft of SCPs, ISCs, non-RSCs, RSCs, and no cell graft. RSCs grafts induced substantial axon regeneration. Left is proximal. Scale bar, 50 μm. **b** Quantification of regenerating axons. RSCs grafts demonstrated the greatest axon-promoting effect among all groups. Non-RSCs grafts revealed statistically more axons than SCPs graft, ISPs graft, and no cell graft. Six rats per group. *P < 0.05 vs. others, **P < 0.05 vs. ISCs, SCPs, and no cell graft, one-way ANOVA with the Tukey–Kramer test. Error bars represent the SEM.

**Fig. 4:**
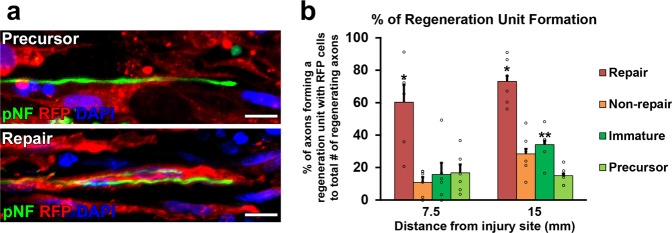
Regeneration unit formation by grafted RSCs. **a** High magnification images of regenerating axons (pNF, green) and grafted cells (RFP, red). Arrowheads indicate regenerating axons, which are tortuous and have growth cone-like morphology. The upper image is a representative example of the lack of a regeneration unit formation by grafted RFP expressing SCPs. RFP expressing SCPs have no association with a regenerating axon. In contrast, the lower image shows that RFP expressing RSCs form a regeneration unit with regenerating axons. Scale bars, 10 μm. Left is proximal. **b** Quantification of regeneration unit formation. Percentages of axons forming a regeneration unit with RFP^+^ cells to total # of regenerating axons were quantified. Grafted RSCs have a great capacity to form regeneration units. Six rats per group. *P < 0.05 vs. others. **P < 0.05 vs. SCPs. One-way ANOVA with the Tukey-Kramer test. Error bars represent the SEM.

Two million of each type of cells in 10 µl phosphate-buffered saline (PBS) were grafted into 25 mm-long acellular regions with crush injuries^20^. As a negative control, 10 µl of PBS alone was injected. Two weeks after cell grafts and injuries, rats were perfused, followed by an immune-histological assessment. Grafted cells survived well and filled up acellular regions in all groups (Fig. 2a, b). Quantification of RFP expressing grafted cells showed that the SCPs and the ISCs groups had greater survival than the RSCs and the nonRSCs groups in proximal regions (Fig. 2c). The grafted ISCs had significantly more Ki-67 positive cells than the RSCs and the nonRSCs in all quantified areas (Fig. 2d, e), whereas the grafted SCPs showed more Ki-67 positive cells than the RSCs and the nonRSCs only in proximal regions. This reduced proliferation of SCPs in the distal regions could be attributed to the fact that the proliferation of SCPs depends on neuregulin released from growing axons^42^. Regarding axon regeneration, the RSCs induced the greatest regeneration among all tested cells at every quantified point (Fig. 3a, b). Next was the nonRSC, which induced significantly more axon regeneration than the other 3 groups at 7.5 mm point from the proximal injury site (Fig. 3b). In contrast, developing SCs did not demonstrate any axon-promoting effect at all, when compared to no cell graft. Further analysis about a regeneration unit, which is a complex of regenerating axon and an SC providing a substrate for growth^43,44^, revealed that a significantly higher % of the RSCs formed regeneration units than other cells (Fig. 4a, b), suggesting the direct effect of the RSCs on axon regeneration. These results indicate that mature SCs but not developing SCs can promote axon regeneration, and the RSCs possess the superior property to directly support axon regeneration by making regeneration units among tested four types of SCs.

### RSCs can promote neurite outgrowth of adult DRG neurons more effectively than SCPs

**Fig. 5:**
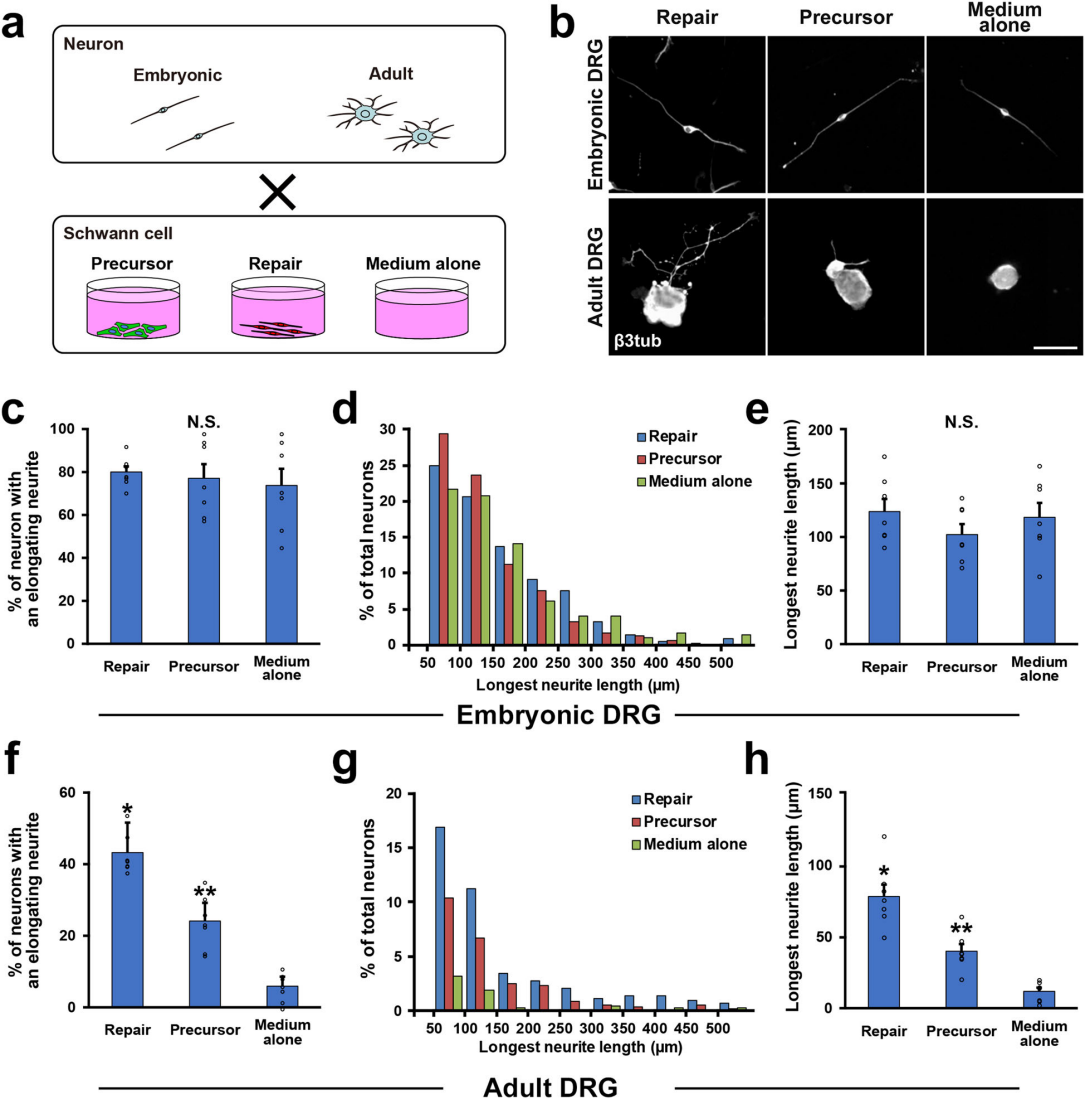
Enhancement of neurite outgrowth of DRG neurons by co-cultured SCs. **a** Schematic diagram of co-culture of DRG neurons and SCs. **b** Representative images of neurite outgrowth of DRG neurons in each condition. Immunolabeling with β3 tubulin depicted neurite elongation. Upper images are embryonic DRG neurons and neurite outgrowth of all three groups is comparable in length. Lower images are adult DRG neurons, showing a neuron co-cultured with RSCs elongated its neurite longer compared to neurons in other conditions. Scale bar, 50 μm. **c** Quantification of % of elongating embryonic DRG neurons. No statistical difference was detected among groups. Seven samples per group. At least 50 neurons were calculated in each sample. **d** Percentage of embryonic DRG neurons with the longest elongating neurite in the categories shown. There is no apparent distribution pattern among groups. **e** Quantification of the longest neurite of embryonic DRG neurons. There is no statistical difference among groups, demonstrating that the presence of co-cultured SCs does not affect neurite outgrowth of embryonic DRG neurons. **f** Quantification of % of elongating adult DRG neurons. RSCs stimulated neurite outgrowth of adult DRG neurons significantly more than SCPs, and the next is SCPs compared to the medium alone. Seven samples per group. At least 50 neurons were calculated in each sample. **g** Percentage of adult DRG neurons with the longest elongating neurite in the categories shown. **h** Quantification of the longest neurite of adult DRG neurons. RSCs promoted the length of neurite outgrowth of adult DRG neurons most among groups and the next is SCPs compared to medium alone. *, **P < 0.05 vs. others. One-way ANOVA with the Tukey–Kramer test. Error bars represent the SEM.

To confirm the finding that only mature but not developing SCs can promote the growth of injured adult axons, we further analyzed the effect of SCs on neurite outgrowth of co-cultured DRG neurons. We compared SCPs with RSCs, because SCPs were expected to promote axon regeneration more than ISCs due to their immaturity^28^ and association with growing axons^45^. Two kinds of DRG neurons were prepared from E14 and adult time points, and they were cocultured with SCPs or RSCs (Fig. 5a). After 24 h of co-culture, embryonic DRG neurons elongate their neurite well regardless of presence or type of SCs (Fig. 5b, c–e), indicating that the intrinsic growth property of embryonic DRG neurons is robust and that their neurite outgrowth is independent of extrinsic factors such molecules provided by cocultured SCs. In contrast, adult DRG neurons rarely elongate their neurites without cocultured SCs (Fig. 5b, f–h), and the cocultured SCs enhanced neurite outgrowth significantly. The effect of RSCs was the most, which was about 97% greater than that of SCPs in the longest neurite length (Fig. 5b, f–h). This result indicates that the growth property of adult DRG neurons is poor but mature SCs can support their neurite outgrowth effectively more than developing SCs.

### Gene expression profile was distinctly different between RSCs and SCPs

**Fig. 6:**
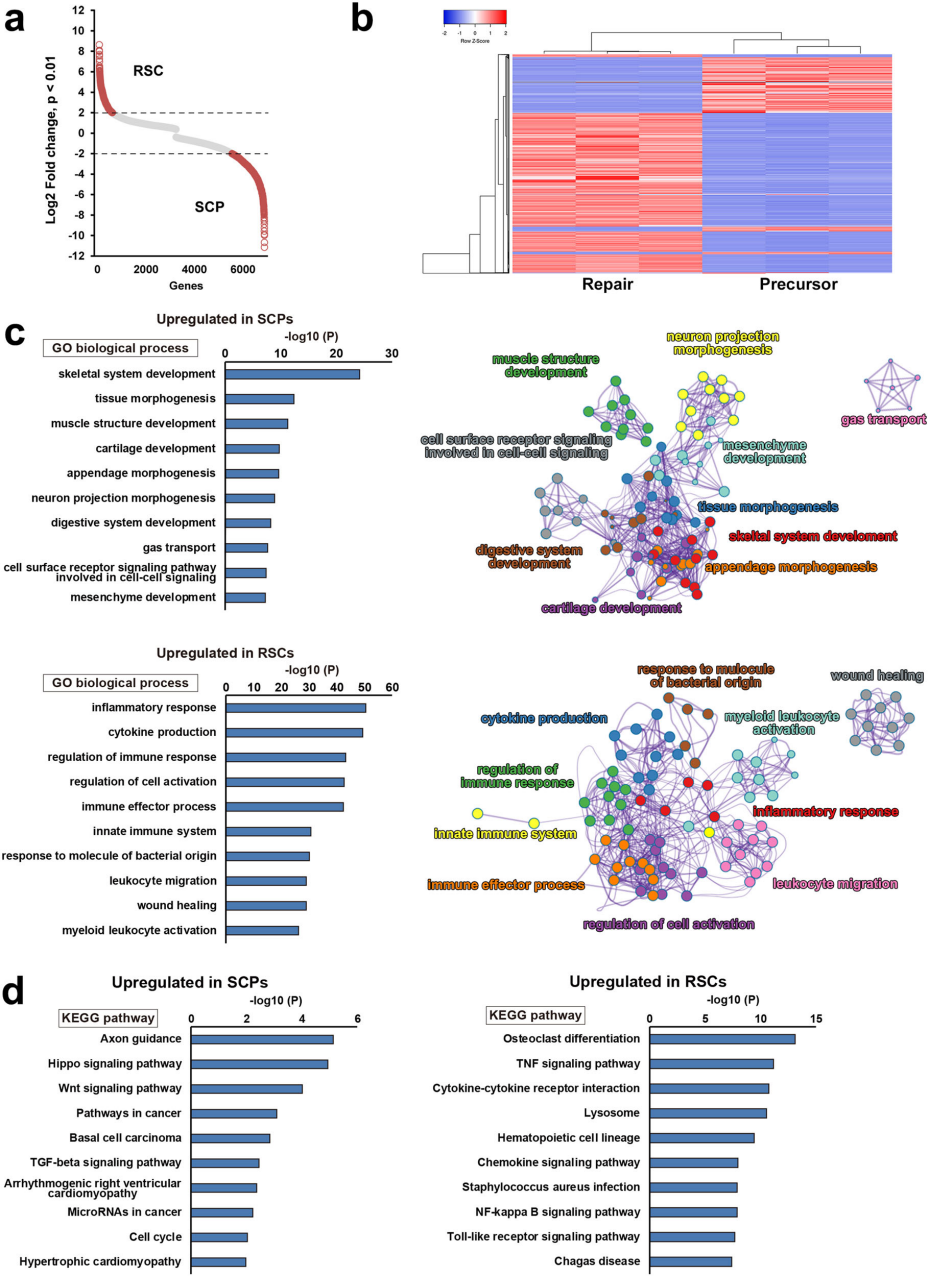
Transcriptome analysis of RSCs and SCPs. **a** Scatter plot analysis for transcript expression levels of significantly upregulated or downregulated genes in RSCs and SCPs. Red circles indicate genes with Log2 fold change over 2 or under −2. Transcript expression levels were significantly different in 6804 genes (*p* < 0.01), and 1933 genes were more than 4-folds changed (*p* < 0.01, log2FC > 2). **b** Heatmap of gene expressions in RSCs and SCPS. They revealed a high degree of similarity between the samples in each group, and gene expression profiles are distinctly different between RSC and SCPs. Red and blue indicate the highest and lowest relative levels of gene expression. **c** Top ten enriched GO terms of biological process and their cluster visualization in SCPs (upper row) and RSCs (lower row). Development-related terms are upregulated in SCPs, whereas inflammation-related terms are upregulated in RSCs. Cluster annotations are shown in each clusters’ color. **d** KEGG pathway analysis in SCPs (left) and RSCs (right). Biological terms are distinctly different between these two cells.

**Fig. 7:**
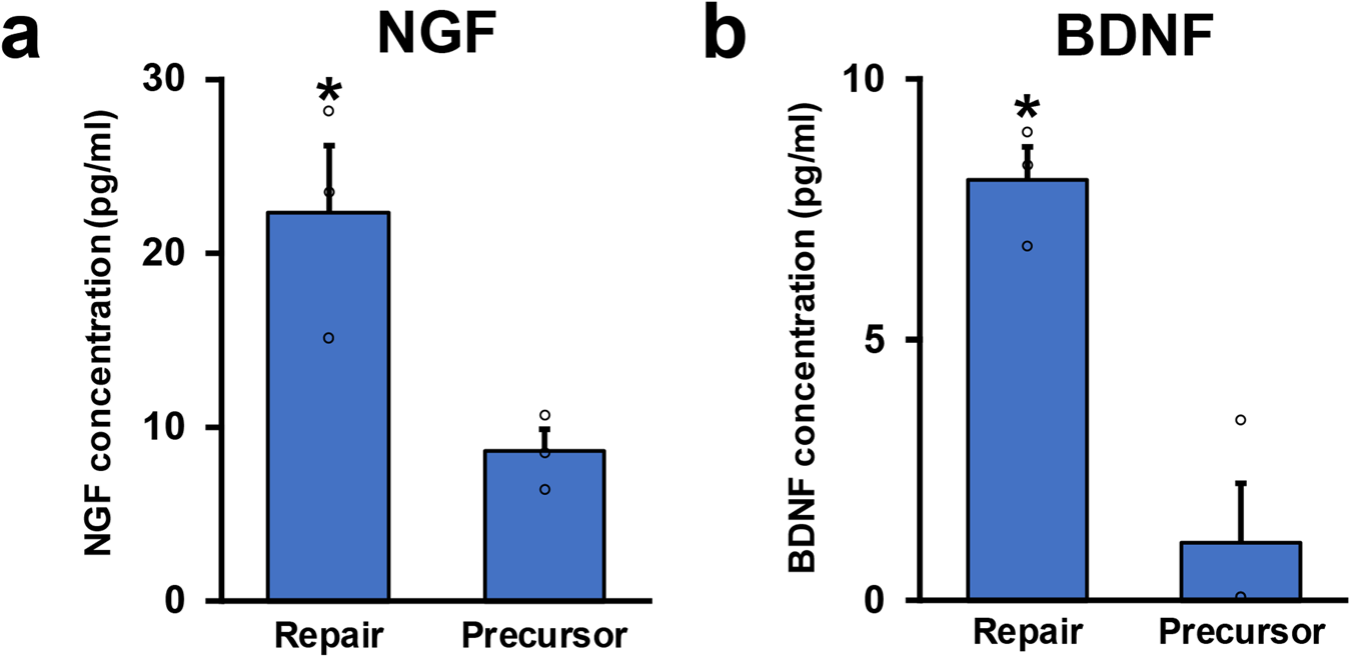
Neurotrophic factor secretion of RSCs and SCPs. **a** NGF concentrations of conditioned medium of RSCs and SCPs. RSCs secrete more than twofold of NGF than SCPs do. Three samples per group. *P < 0.05; Student’s *t*-test. Error bars represent the SEM. **b** BDNF concentrations of conditioned medium of RSCs and SCPs. RSCs secrete more than fivefold of BDNF than SCPs do. Three samples per group. *P < 0.05; Student’s *t*-test. Error bars represent the SEM.

To explore the molecular mechanisms underlying the marked difference of axon-promoting effects between the RSCs and the SCPs, we performed their transcriptome analysis by RNA-seq. Transcript expression levels were significantly different in 6804 genes (*p* < 0.01), and 1933 genes were more than 4-folds changed (*p* < 0.01, log2FC > 2), clearly indicating that gene expression profiles of the RSCs and the SCPs are distinctly different (Fig. 6a, b). Additional analysis by gene ontology (GO) showed that several biological features were also significantly different. The top seven genes enriched in the SCPs were development and morphogenesis-related ones but not repair and inflammation-related ones, whereas these in the RSCs were inflammatory response, cytokine production, and regulation of immune response (Fig. 6c), that are known to involve in repair processes after PNI^44,46–48^. Kyoto Encyclopedia of Genes and Genomes (KEGG) pathway analysis also identified axon guidance as to the most upregulated pathway in the SCPs (Fig. 6d), as expected in glial cells at the developing stage. Other well-known pathways in developing SCs, Hippo, and Wnt signaling pathways were also upregulated^28,49,50^. In contrast, in the RSCs, upregulated pathways were osteoclast differentiation, TNF signaling, and Toll-like receptor signaling pathways (Fig. 6d), which are related to the AP-1(Jun/Fos) transcription factor that is critical for the repair function of the RSCs^51,52^. Furthermore, a considerable number of genes related to basement membrane components such as collagens, laminins, and fibronectins^53^ are differently expressed (Supplementary Fig. 2). These findings indicate that there are rarely shared molecular features between the SCPs and the RSCs and that the molecular profile of the SCPs is exclusively related to development but not the repair process to which the molecular profile of the RSCs is mainly related. Lastly, we quantified the secretion of two axon-promoting neurotrophic factors, nerve growth factor (NGF) and brain-derived neurotrophic factor (BDNF)^54^ in the SCPs and the RSCs. Notably, the RSCs produced significantly more NGF and BDNF than the SCPs (Fig. 7), supporting the finding of the marked difference of axon-promoting effect of these SCs at the protein level.

## Discussion

The current study demonstrated that the capacity of SCs to promote axon regeneration after PNI totally depended on their developmental stage. Developing SCs such as SCPs and ISCs failed to promote axon regeneration, whereas mature SCs could induce axon regeneration. This is quite notable because there are several reasons to expect developing SCs to promote axon regeneration after PNI. First, many literatures report that developing cells have a therapeutic effect on tissue repair or regeneration^8,9,12,55–57^. Second, in CNS injury models, grafts of developing cells induced axon regeneration^10,30,58^. Third, when developing neurons extend their axons, neighboring immature glial cells support them^38,45,59,60^. Fourth, because developing cells are generally more proliferative and resilient than mature cells, they could survive better when grafted. Lastly, developing cells differentiate in the graft environment. Since they are plastic, they might adopt the injured environment and differentiate into more reparative phenotype^34,61–65^. Regardless of these reasonable facts, developing SCs has no axon promoting effect at all after PNI, clearly indicating that developing SCs are specialized in supporting developing axons but not injured adult axons and that the extrinsic mechanism of regenerating axons differ from that of developing axons.

Developing SCs showed interesting behavior after grafting in the current study. The number of survived SCPs was smaller in more distal areas than the proximal area (Fig. 2c). In development, the survival of SCPs but not ISCs depends on secreted molecules such as neuregulin^66^ from neighboring growing axons^42,67^. Accordingly, this interesting behavior is assumed to be the result that regenerating axons secrete soluble molecules stimulating SCPs like developing axons^68,69^ and that only SCPs close to regenerating axons can survive after grafting by receiving stimulation of secreting molecules from axons. This finding suggests that prepared SCPs still retain the initial phenotype even after grafting into the injured environment.

Accumulated evidence shows that axon regeneration requires both intrinsic and extrinsic mechanisms^70–74^. Based on the result of the co-culture experiment (Fig. 5) and others^43^, adult DRG neurons need extrinsic support from SCs for neurite outgrowth. In contrast, developing DRG neurons demonstrated substantial neurite outgrowth independently from co-cultured SCs (Fig. 5c, e), suggesting that developing DRG neurons have a great intrinsic growth capacity and require no extrinsic support from SCs. Indeed, developing SCs contribute to axonal sorting and maintenance but not to axonal extension^60,75^, supporting this observation. The extrinsic mechanism of axon regeneration consists of secreted factors as well as contact-mediated factors^43,76^. Regarding the former factors, SCPs do not secrete a comparable amount of neurotrophic factors to RSCs (Fig. 7). About the latter factors, their expression profile of collagens, laminins, and fibronectins, which are major extracellular matrix (ECM) molecules associated with basement membrane^53,77^ was quite different from that of RSCs (Supplementary Fig. 2), and developing SCs couldn’t form many regeneration units (Fig. 4b)^78^. Although the exact function of ECM molecules on axon regeneration is not fully understood, some type of collagen and laminin is reported to have critical roles in axon regeneration^79,80^. The obtained results suggest that developing SCs generate less ECM necessary for axon regeneration than mature SCs, and these facts explain at least partially the reason why developing SCs failed to promote axon regeneration.

In the current study, we employed a 2-week time point to assess the axon promoting effects of grafted cells, because this time point was originally defined as optimal to compare the axon promoting effects in this experimental model^20^. At later time points, this model has less sensitivity to detect the difference of axon promoting effects, because more axons regenerate and more host SCs migrate into acellular region. Regarding the therapeutic effects of each SCs, we did not perform a functional assessment, because it does not necessarily reflect the ability to promote axon regeneration of grafted SCs. Functional recovery after PNI requires axon regeneration and then remyelination. However, the capacity of each SCs to promote remyelination remains unclear. Myelination-related genes are differentially expressed in each SCs^81,82^, indicating that their remyelinating effects after PNI would vary. In addition, the extent of proliferation is different among each SCs (Fig. 2e). Although the ability of grafted SCs to promote axon regeneration is critical, multiple aspects of SCs will affect therapeutic effects induced by the graft of SCs.

The current study revealed that molecular profiles of SCPs and RSCs were distinctively different (Fig. 6), supporting the recent idea that RSCs are not dedifferentiated SCs like developing SCs^35,44^. In detail, SCPs upregulate guidance molecules and pathways related to development, whereas RSCs upregulate cytokine production, inflammation-related molecules, and pathways, exactly reflecting the environment where SCs located (Fig. 6). SCPs are surrounded by immature cells and little inflammation, whereas RSCs are in the center of inflammation consisting of debris and immune cells^47,83–86^. This molecular comparison data provides the basis for analyzing the characteristics of RSCs in addition to the previous analysis^48^.

Based on the current findings, RSC is a potent candidate as a graft material for cell therapy of PNI, especially compared to nonRSCs. However, previous attempts of SC grafts for PNI used nonRSCs and there was little information on their molecular and cellular profiles^14,16–18^. SCs were prepared from the patient’s intact sural nerve or intact rat sciatic nerve and expanded several times. In those studies, if RSCs were used instead of nonRSCs, more regeneration could have been induced. For clinical translation, it is necessary to establish the protocol to generate RSCs from nonRSCs and maintain their therapeutic effects. Ideally, the generation of RSCs from pluripotent stem cells or allogenic cells is suitable for a practical clinical application, since no sacrifice of the nerve is required.

In rodents, SCs start to change their transcriptional pattern into repair phenotype in several hours after PNI^48^, and by one to four weeks after PNI, they dramatically change its morphology through demyelination, elongation, and branching^87^. These steps undergo smoothly in young mice, however, in aged mice, phenotypic change of SCs is impaired, resulting in poor axon regeneration^72^. Based on these findings with the current findings, acceleration of phenotypic change from nonRSCs into RSCs is a therapeutic target for PNI.

In conclusion, RSCs demonstrated the greatest axon-promoting effects among four types of SCs in different developmental stages. Unlike CNS, developing glia had no significant effects on axon regeneration. Future therapeutic strategies include implantation of RSCs with an establishment of conversion method from nonRSC to RSC and an identification of key molecules expressed by RSCs for axon regeneration.

## Methods

### Animals

Adult LEWIS rats (Wild-type, Charles River Laboratories Japan, Inc.) were used in all experiments. Their body weight ranged from 155 to 211 g with an average of 185 g. Graft cells were prepared from syngeneic adult LEW-Tg (Gt(ROSA)26Sor-DsRed*)7Jmck rats that ubiquitously express the DsRed monomer driven by the gene trap ROSA 26 promoter, supplied by the National BioResource Project (Kyoto University, Kyoto, Japan). The study protocol was approved by the local ethical committee of Hokkaido University. Animals had free access to food and water throughout the study. For animal anesthesia, a mixture of ketamine (75–100 mg/kg, KETALAR^®^, Daiichi Sankyo Propharma Corporation, Tokyo, Japan) and medetomidine (0.5 mg/kg, DOMITOR^®^, Orion Corporation, Espoo, Finland) was administered by intraperitoneal injection.

### SC preparation

All grafted cells used in this study were prepared from transgenic LEWIS rats that ubiquitously expressed RFP and that were syngeneic to wild-type LEWIS rats. SCPs and ISCs were harvested using a modified protocol described previously^38,88^. In brief, E14 and E18 Embryos were harvested from time-mated pregnant females, and bilateral sciatic nerves and brachial plexus were dissected using fine forceps (Fine Science Tools, No.11252-202). Then, dissected nerves were transferred to an enzymatic digestion medium containing 0.1% collagenase I (Sigma-Aldrich) and 0.125% trypsin in Dulbecco’s modified Eagle medium (DMEM)/Ham’s F-12 (DMEM/F12, Wako, Osaka, Japan). After incubation for 30 min at 37 °C, nerves were mechanically dissociated by pipetting 30 times in a 1 ml SC culture medium, which consists of DMEM/Ham’s F-12 supplemented with 10% fetal bovine serum (FBS), 1% GlutaMAX (Thermo Fisher Scientific, Waltham, MA), and 1% penicillin-streptomycin (PS, Thermo Fisher Scientific). RSCs and non RSCs were prepared from intact sciatic nerves and distal segments of transected sciatic nerves of 8–10 week old rats according to a modified protocol^89^. Briefly, sciatic nerves running from the sciatic notch or the transection site to the end of the femur were dissected, cut into 1- to 2-mm pieces using micro-scissors after removal of the epineurium, and transferred to enzymatic digestion medium containing 1% collagenase I and 0.125% trypsin in DMEM/F12. After incubation for 1 h at 37 °C, tissues were mechanically dissociated by pipetting 30 times in a 1 ml SC culture medium. To remove myelin debris, cell suspension of non RSCs were resuspended in 10 ml SC culture medium, and mixed with 8 ml Percoll plus (GE Healthcare, Chicago, IL), 2 ml 10× HBSS (Wako, Osaka, Japan), 10 ml 1× HBSS, and centrifuged 30 min in 4 °C using fixed angle centrifuge rotors. Ten ml solution at the bottom was gently taken and washed with 1× HBSS. Lastly, to remove adhesive cells such as fibroblasts and macrophages, cells were seeded in 75 cm^2^ non-coated flasks for 30 min, and cells floating in culture medium were used for experiments. Cell viability, which was assessed with trypan blue (Life Technologies, Grand Island, NY), was within the range of 92–99% for all cell preparations. For characterization, cells were cultured on poly-l-lysine (PLL, Sigma-Aldrich) and laminin (Sigma-Aldrich) coated 24-well plates at a density of 1.0 × 10^5^ cells/cm^2^ with the SC culture medium. For grafting and RNA extraction, prepared cells were resuspended in PBS (Thermo Fisher Scientific). For coculture experiment or ELISA analysis, cells were resuspended DMEM/F12 supplemented with 2% B27 (Thermo Fisher Scientific), Heregulin (20 ng/ml, Peprotech), 1% GlutaMAX, and 1% PS.

### Surgical procedures

A recently developed experimental model dedicated to assessing the efficacy of grafted cells on axon regeneration was used^20^. Briefly, the sciatic nerve was exposed and received 2 crush injuries made by micro-mosquito forceps. The proximal injury was just distal to the sciatic notch, and the distal injury was 25 mm distal to it. For making decellularization area, this 25-mm long area between two injury sites was subject to repeated frozen by liquid nitrogen and spontaneous thaw at room temperature five times. To mark the injury site, a stay suture was placed at the epineurium just next to the injury site. A total of 30 rat sciatic nerves were divided into the following five groups, (1) SCP grafts, (2) ISC grafts, (3) nonRSC grafts, (4) RSC grafts, and (5) no cell graft. One million cells in 10 µl PBS were grafted into the decellularized area by four injections through a 34 gauge needle of NanoFil syringe (World Precision Instruments, Sarasota, FL) in cell grafts groups, and 10 µl PBS alone were injected using the same method in the no cell graft group.

### Coculture of DRG neurons and SCs

Embryonic DRGs and adult lumber DRGs were dissected from E14 and 10–12 weeks old wild-type LEWIS rats. Embryonic DRGs were incubated in 0.1% collagenase type XI(Sigma-Aldrich) for 1 h at 37 °C, and adult DRGs were incubated in 0.5% collagenase type XI solution for 1 h at 37 °C. After enzymatic digestion, they were mechanically dissociated by pipetting 30 times in a 1 ml medium, consisting of DMEM/Ham’s F-12 supplemented with 2% B27, 1% GlutaMAX, and 1% PS.^8,9,90^ Embryonic and adult DRG neurons were cocultured with SCPs or RSCs. SCs were seeded at a density of 5.0 × 10^4^ cells/cm^2^ on the 48-well plate coated with PLL and laminin. Three hours later, 5.0 × 10^3^ DRG neurons/cm^2^ were placed in the wells with the neuron culture medium. Twenty-four hours later, cells were fixed with 4% paraformaldehyde (PFA, Nacalai Tesque Inc., Kyoto, Japan) in 0.1% phosphate buffer (PB). Neurite outgrowth was quantified as described previously^43^. Images were taken by an all-in-one fluorescent microscope (BZ-X710, Keyence, Osaka, Japan) using a 20× objective lens. Neurites were traced and measured by ImageJ^91^ with plugin software, NeuronJ, as described previously^71,92^. To avoid the effect of neuron-neuron interaction, if their neurites touched neurites of other neurons, these neurons were excluded from the analysis. Fifty micrometer or longer neurite was defined as an elongating neurite^93^. At least 50 neurons were randomly selected per well, and the % of neurons with elongating neurites and the averages of longest neurites were calculated. Experiments were repeated three times and a total of six wells per condition were obtained.

### Immunohistochemistry and immunocytochemistry

For immunohistochemistry, sciatic nerves were dissected after perfusion with 4% PFA in 0.1% PB, followed by overnight fixation with 4% PFA at 4 °C. On the following day, nerves were transferred into 30% sucrose in 0.1% PB and stored until sectioning. When sectioning a nerve, a small cut was made at the injury site to mark the injury site. Nerves were sagittally sectioned using cryostat at 10-μm intervals and directly mounted on 10 slides in order. For immunocytochemistry, cultured cells were fixed with 4% PFA in 0.1% PB for 15 min. Fixed cells or sections were incubated overnight with primary antibodies against RFP (1:200, goat from Sicgen, Portugal), pan neurofilament (pNF, 1:1000, mouse from Biolegend, San Diego, CA), S100β (1:200, rabbit from Abcam, Cambridge, UK), Ki67 (1:500, rabbit from GeneTex, Irvine, CA), β3 tubulin (1:1000, rabbit and mouse from Biolegend), Nestin (1:500, mouse from BD Bioscience, Franklin Lakes, NJ), Sox2 (1:500, rabbit from Merck Millipore, Burlington, MA), and Sox10 (1:100, goat from R&D systems, Minneapolis, MN) at 4 °C. Then, after washing with Tris-buffered saline, sections were incubated in Alexa 488, 594, or 647 conjugated to donkey secondary antibodies (1:1000, Jackson Immunoresearch, West Grove, PA) and DAPI for 1 h at room temperature.

### Quantification

Three consecutive sections from the middle part of the nerve were used for quantification of in vivo studies. The total number of RFP expressing cells were identified by the presence of DAPI surrounded by RFP immunoreactivity at high magnification image, quantified in a 100-µm wide region at three points, which were 7.5, 15, 22.5 mm distal to the proximal injury site, and divided by the total quantified area as the density of grafted cells. For quantifying the proliferating grafted cells, the RFP and Ki67 double-immunoreactive cells were counted in the same manner. Axon regeneration rate was quantified as described previously^20^. Briefly, lines perpendicular to sections were set at points 7.5, 15, 22.5, and 30 mm distal and just proximal to the injury site. The numbers of pNF-labeled axons crossing each line were quantified. For normalization, the sum of the axon numbers of three sections was divided by the sum of the length of each line as an axon density. To calculate the percentage of axon regeneration, the axon density of each point was divided by the density at the uninjured site, which was 1.5 mm proximal to the injury site. To evaluate the three-dimensional relationship between axons and grafted cells, triple stained sections for pNF, RFP, and DAPI were imaged at points 7.5 and 15 mm distal to the injury site in which regenerating axons are observed in all samples with a confocal laser microscope (FV-1000, Olympus, Tokyo, Japan) at the 1000× magnification. The number of axons that have a close association with graft cells was divided by the total number of axons as the % of regeneration unit formation.

### RNA-seq

RNA was extracted from SCPs and RSCs using TRIzol (Thermo Fisher Scientific, Waltham, MA) and RNeasy plus mini kit (Qiagen, Netherland) using the manufacturer’s instructions. Total RNA integrity and quality were assessed with Aglient 2100 bioanalyzer (Aglient Technologies, Santa Clala, CA, USA), and samples over 1 μg which had an RNA integrity number greater than 7 were used for RNA-seq. Each library was generated using the TruSeq Strandard mRNA Sample Preparation Kit (Illumina, Inc., San Diego, CA, USA), and paired-end reads (100 bp) were obtained by Illumina NovaSeq 6000 (Illumina, Inc). Reads were mapped by alignment to rattus norvegicus genome rn6 and analyzed by TopHat, Cuff links, and Cuffdiff^94^ Genes with *p* value < 0.01 and log2FC > 2 were defined as differentially expressed genes (DE genes). GO and KEGG (Kyoto Encyclopedia of Genes and Genome) pathway analyses were performed using DAVID (https://david.ncifcrf.gov/) databases and metascape (https://metascape.org/gp/index.html#/main/step1). Heatmap was made using Heatmapper (http://www.heatmapper.ca/expression/). Clusters of biological processes were visualized by prefuse force-directed layout using Cytoscape (https://cytoscape.org).

### Enzyme-linked immunosorbent assay (ELISA)

SCPs and RSCs were seeded at a density of 2.0 × 10^5^/well on the 24-well plate coated with PLL and laminin and cultured in 1.5 ml of culture media mentioned above. Concentrations of NGF and BDNF were measured using Rat NGF/BDNF ELISA kit (Abcam, Cambridge, UK) following the manufacturer’s instruction (*N* = 3/group). The absorbance was read at 450 nm using Benchmark-Plus Reader (BIO-RAD, CA).

### Statistical analysis

The normality of the data distribution was assessed with the Shapiro–Wilk test. Multiple-group comparisons were made with Kruskal–Wallis analysis of variance and the Tukey–Kramer test, and two-group comparisons were made with the unpaired two-tailed Student’s *t*-test. All analyses were performed with JMP Pro 14.0 software (SAS Institute, Cary, NC) with a pre-specified significance level of 95%. Data are presented as the mean ± standard error of the mean (SEM).

### Reporting summary

Further information on research design is available in the Nature Research Reporting Summary linked to this article.

## Supplementary information

Supplementary Figure

REPORTING SUMMARY

## Data Availability

The datasets generated during and/or analyzed during the current study are available from the corresponding author on reasonable request. Sequence data that support the findings of this study are deposited in Gene Expression Omnibus under the accession code GSE188399.
