# Supplementary material for: Mature but not developing Schwann cells promote axon regeneration after peripheral nerve injury
Source: NPJ Regen Med. 2022 Jan 28;7:12. doi: 10.1038/s41536-022-00205-y (PMC8799715; doi:10.1038/s41536-022-00205-y)
Supplement: Supplementary file 1 — Supplementary Figure [file 41536_2022_205_MOESM1_ESM.pdf]

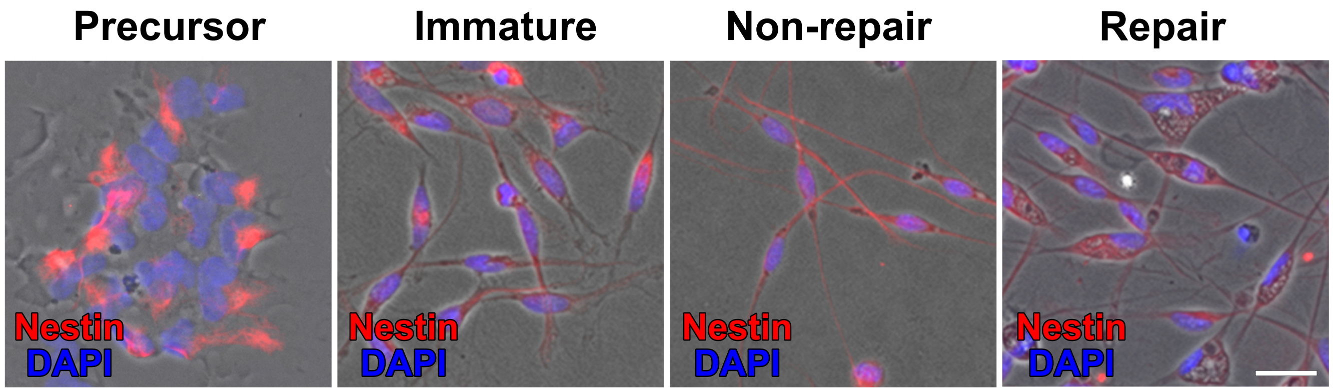

Supplementary Figure 1: Nestin expression of prepared SCs

Phase-contrast images and nestin immunolabeling of prepared SCs. Nestin is expressed in all type of SCs. Scale bars: 20 μm.

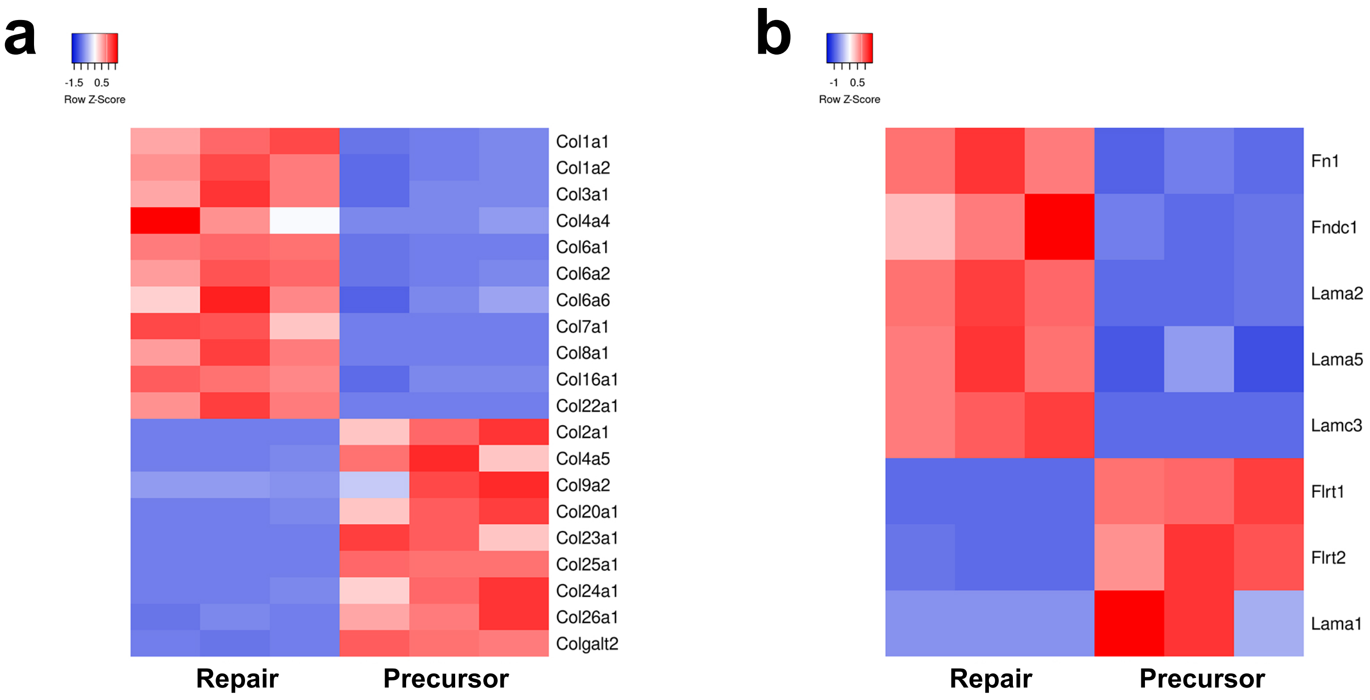

Supplementary Figure 2: Expression patten of genes related to extra-cellular matrix production

Heatmaps of genes related to collagen (a), laminin and fibronectin (b) with significant different expression between RSCs and SCPs excerpted from Figure 6b. Gene expression profiles of extra-cellular matrix are distinctly different between RSC and SCPs.
