# Supplementary material for: Mature but not developing Schwann cells promote axon regeneration after peripheral nerve injury
Source: NPJ Regen Med. 2022 Jan 28;7:12. doi: 10.1038/s41536-022-00205-y (PMC8799715; doi:10.1038/s41536-022-00205-y)
Supplement: Supplementary file 2 — REPORTING SUMMARY [file 41536_2022_205_MOESM2_ESM.pdf]

nature portfolio

Corresponding author(s): Ken Kadoya

Last updated by author(s): Dec 4, 2021

Reporting Summary

Nature Portfolio wishes to improve the reproducibility of the work that we publish. This form provides structure for consistency and transparency in reporting. For further information on Nature Portfolio policies, see our Editorial Policies and the Editorial Policy Checklist.

Statistics

For all statistical analyses, confirm that the following items are present in the figure legend, table legend, main text, or Methods section.

n/a	Confirmed	
☐	☒	The exact sample size (n) for each experimental group/condition, given as a discrete number and unit of measurement
☐	☒	A statement on whether measurements were taken from distinct samples or whether the same sample was measured repeatedly
☐	☒	The statistical test(s) used AND whether they are one- or two-sided<br>*Only common tests should be described solely by name; describe more complex techniques in the Methods section.*
☒	☐	A description of all covariates tested
☐	☒	A description of any assumptions or corrections, such as tests of normality and adjustment for multiple comparisons
☐	☒	A full description of the statistical parameters including central tendency (e.g. means) or other basic estimates (e.g. regression coefficient) AND variation (e.g. standard deviation) or associated estimates of uncertainty (e.g. confidence intervals)
☐	☒	For null hypothesis testing, the test statistic (e.g. F, t, r) with confidence intervals, effect sizes, degrees of freedom and P value noted<br>*Give P values as exact values whenever suitable.*
☒	☐	For Bayesian analysis, information on the choice of priors and Markov chain Monte Carlo settings
☒	☐	For hierarchical and complex designs, identification of the appropriate level for tests and full reporting of outcomes
☒	☐	Estimates of effect sizes (e.g. Cohen's d, Pearson's r), indicating how they were calculated

Our web collection on statistics for biologists contains articles on many of the points above.

Software and code

Policy information about availability of computer code

Data collection	Images were collected using all-in-one flurorescent microscope (BZ-X710, Keyence, Osaka, Japan) and confocal laser microscope (FV-1000, Olympus, Tokyo, Japan). RNA sequencing was performed on the Illumina NovaSeq 6000(Illumina, Inc). In enzyme-linked immunosorbent assay, the absorbance was read using Benchmark-Plus Reader (BIO-RAD, CA).
Data analysis	Statistical analysis was conducted with JMP Pro 14.0 software (SAS Institute, Cary, NC)<br>Images were processed using ImageJ 1.51r software (https://imagej.nih.gov/ij/index.html).<br>In RNA-seq, reads were mapped by alignment to rattus norvegicus genome rn6 and analyzed by TopHat, Cuff links, and Cuffdiff.<br>Data analysis were performed by following resourses:<br>DAVID (https://david.ncifcrf.gov/)<br>Heatmapper (http://www.heatmapper.ca/expression/)<br>Metascape (https://metascape.org/gp/index.html#/main/step1)<br>Cytoscape (https://cytoscape.org)

For manuscripts utilizing custom algorithms or software that are central to the research but not yet described in published literature, software must be made available to editors and reviewers. We strongly encourage code deposition in a community repository (e.g. GitHub). See the Nature Portfolio guidelines for submitting code & software for further information.

Data

Policy information about availability of data

All manuscripts must include a data availability statement. This statement should provide the following information, where applicable:

- Accession codes, unique identifiers, or web links for publicly available datasets
- A description of any restrictions on data availability
- For clinical datasets or third party data, please ensure that the statement adheres to our policy

Sequence data that support the findings of this study are deposited in Gene Expression Omnibus under the accession code GSE188399.
Other raw data that supports the findings of the current study are available from the corresponding author upon reasonable request.

Field-specific reporting

Please select the one below that is the best fit for your research. If you are not sure, read the appropriate sections before making your selection.

☒ Life sciences ☐ Behavioural & social sciences ☐ Ecological, evolutionary & environmental sciences

For a reference copy of the document with all sections, see nature.com/documents/nr-reporting-summary-flat.pdf

Life sciences study design

All studies must disclose on these points even when the disclosure is negative.

Sample size	For the cell graft investigation, 30 rats(6 rats per group) were used as host. For coculture of DRG neurons and Schwan cells, we used 7 wells per each condition. For Transcriptome analysis and enzyme-linked immunosorbent assay, we prepared 3 sets of Schwann cells per each group. No statistical methods were used to pre-determine sample size. But, our sample sizes are similar to the following previous publications.<br><br>Veselina Petrova, et al. Protrudin functions from the endoplasmic reticulum to support axon regeneration in the adult CNS. Nature Communications volume 11, Article number: 5614 (2020<br>Poplawski GHD, et al. Adult rat myelin enhances axonal outgrowth from neural stem cells. Sci Transl Med. 2018 May 23;10(442):eaal2563<br>Mark Anderson, et al. Required growth facilitators propel axon regeneration across complete spinal cord injury. Nature. 2018 Sep;561(7723):396-400.<br>Leibinger M, et al. Boosting CNS axon regeneration by harnessing antagonistic effects of GSK3 activity. Proc Natl Acad Sci U S A. 2017 Jul 3;114(27):E5454-E5463.
Data exclusions	No data were excluded from the analyses.
Replication	All in vivo studies assessing axon regeneration effects had control groups treated with PBS. All animal studies consisted of over 3 different sets of experiments, showing reproducibility. All in vitro studies assessing neurite extension effects had control groups treated with the same medium. RNA-seq measurement and enzyme-linked immunosorbent assay consisted of 3 sets of experiments.
Randomization	Animals were assigned randomly to control and experimental groups.
Blinding	Group identity was not blinded when quantification was performed.

Reporting for specific materials, systems and methods

We require information from authors about some types of materials, experimental systems and methods used in many studies. Here, indicate whether each material, system or method listed is relevant to your study. If you are not sure if a list item applies to your research, read the appropriate section before selecting a response.

Materials & experimental systems

n/a	Involved in the study	
☐	☒	Antibodies
☒	☐	Eukaryotic cell lines
☒	☐	Palaeontology and archaeology
☐	☒	Animals and other organisms
☒	☐	Human research participants
☒	☐	Clinical data
☒	☐	Dual use research of concern

Methods

n/a	Involved in the study	
☒	☐	ChIP-seq
☒	☐	Flow cytometry
☒	☐	MRI-based neuroimaging

Antibodies

Antibodies used	RFP (1:200, goat from Sicgen, AB0040-200, Portugal)<br>pan neurofilament (1:1000, mouse from BioLegend, 837904, San Diego, CA)<br>S100β (1:200, rabbit from Abcam, ab52642, Cambridge, UK)<br>Ki67(1:500, rabbit from GeneTex, gtx16667, Irvine, CA)<br>β3 tubulin(1:1000, rabbit from Covance, PRB-435P, Princeton, NJ)<br>Nestin(1:500, mouse from BD Bioscience, BD556309, Franklin Lakes, NJ)<br>Sox2(1:500, rabbit from Merck Millipore, AB5603, Burlington, MA)<br>Sox10 (1:100, goat from R&D systems, AF2864, Minneapolis, MN)
Validation	The validation of antibodies for species and application was described in the respective manufacturers or validated in previous paper like below.<br><br>RFP, pan neurofilament, S100β: Endo T, et al. A Novel Experimental Model to Determine the Axon-Promoting Effects of Grafted Cells After Peripheral Nerve Injury. Front Cell Neurosci. 2019 Jun 28;13:280<br>β3 tubulin, Ki67: verified by manufacture<br>Nestin: Widera D, et al. Schwann Cells Can Be Reprogrammed to Multipotency by Culture. Stem Cells and Development 2011;20(12):2053-2064., Kadoya K, et al. Spinal cord reconstitution with homologous neural grafts enables robust corticospinal regeneration. Nat Med 2016;22(5):479-487.<br>Sox2: Lee S-G, et al. Naked Mole Rat Induced Pluripotent Stem Cells and Their Contribution to Interspecific Chimera. Stem Cell Reports 2017;9(5):1706-1720.<br>Sox10: Fazal SV, et al. Graded elevation of c-Jun in Schwann cells in vivo: gene dosage determines effects on development, re-myelination, tumorigenesis and hypomyelination. J Neurosci 2017;37(50):12297-12313.

Animals and other organisms

Policy information about studies involving animals; ARRIVE guidelines recommended for reporting animal research

Laboratory animals	Details on the rats used in this study are included in the Methods section of this manuscript. Adult WT LEWIS rats (Charles River Laboratories Japan, Inc.) were used in all experiments. Graft cells were prepared from syngenic LEWIS-Transgenic(Tg)(Gt(ROSA)26Sor-luc)11Jmsk rats supplied by the National BioResource Project (Kyoto University).
Wild animals	The study did not involve wild animals.
Field-collected samples	The study did not involve samples collected from the field.
Ethics oversight	All animal procedures were performed in accordance with the guide for the care and use of laboratory animals and were approved by the local ethical committee of the Hokkaido University.

Note that full information on the approval of the study protocol must also be provided in the manuscript.
